# Elevated levels of circulating microRNA-200 family members correlate with serous epithelial ovarian cancer

**DOI:** 10.1186/1471-2407-12-627

**Published:** 2012-12-28

**Authors:** Casina WS Kan, Michael A Hahn, Gregory B Gard, Jayne Maidens, Jung Yoon Huh, Deborah J Marsh, Viive M Howell

**Affiliations:** 1 Hormones and Cancer Division, Kolling Institute of Medical Research, University of Sydney E25, Royal North Shore Hospital, St Leonards, NSW, 2065, Australia; 2 Department of Obstetrics and Gynecology, Royal North Shore Hospital, St Leonards, Australia

**Keywords:** Biomarker, Serum microRNA, miR-200, miR-103, Serous ovarian cancer

## Abstract

**Background:**

There is a critical need for improved diagnostic markers for high grade serous epithelial ovarian cancer (SEOC). MicroRNAs are stable in the circulation and may have utility as biomarkers of malignancy. We investigated whether levels of serum microRNA could discriminate women with high-grade SEOC from age matched healthy volunteers.

**Methods:**

To identify microRNA of interest, microRNA expression profiling was performed on 4 SEOC cell lines and normal human ovarian surface epithelial cells. Total RNA was extracted from 500 μL aliquots of serum collected from patients with SEOC (*n* = 28) and age-matched healthy donors (*n* = 28). Serum microRNA levels were assessed by quantitative RT-PCR following preamplification.

**Results:**

microRNA (miR)-182, miR-200a, miR-200b and miR-200c were highly overexpressed in the SEOC cell lines relative to normal human ovarian surface epithelial cells and were assessed in RNA extracted from serum as candidate biomarkers. miR-103, miR-92a and miR -638 had relatively invariant expression across all ovarian cell lines, and with small-nucleolar C/D box 48 (RNU48) were assessed in RNA extracted from serum as candidate endogenous normalizers. No correlation between serum levels and age were observed (age range 30-79 years) for any of these microRNA or RNU48. Individually, miR-200a, miR-200b and miR-200c normalized to serum volume and miR-103 were significantly higher in serum of the SEOC cohort (*P* < 0.05; 0.05; 0.0005 respectively) and in combination, miR-200b + miR-200c normalized to serum volume and miR-103 was the best predictive classifier of SEOC (ROC-AUC = 0.784). This predictive model (miR-200b + miR-200c) was further confirmed by leave one out cross validation (AUC = 0.784).

**Conclusions:**

We identified serum microRNAs able to discriminate patients with high grade SEOC from age-matched healthy controls. The addition of these microRNAs to current testing regimes may improve diagnosis for women with SEOC.

## Background

Epithelial ovarian cancer (EOC) is the most lethal gynecological malignancy, with serous epithelial ovarian cancer (SEOC) being the most common subtype. Five-year survival for ovarian cancer is approximately 86% when the tumor is localized to the ovary; however, the majority of ovarian cancers are diagnosed at an advanced stage where 5-year survival falls below 25% [1]. This disparity in survival between early and late stage diagnosis emphasizes the need to improve early detection of EOC. Currently, diagnosis may involve physical examination, transvaginal ultrasound and measurement of the serum glycoprotein Cancer Antigen 125 (CA-125). CA-125 has been reported to detect less than 50% of early stage disease, can be elevated in other conditions including pregnancy, menstruation and endometriosis, as well as other cancers and overall is less reliable than imaging for the diagnosis of ovarian cancer [2,3]. Other markers include the glycoprotein human epididymis protein 4 (HE4) [4] and OVA1 (Quest Diagnostics, Madison, NJ, USA), a multivariate index assay comprised of a panel of 5 markers, Transthyretin, Apolipoprotein A-1, beta2-Microglobulin, Transferrin and CA-125 [5,6]. Although OVA1 is successful in determining malignancy for preoperative evaluation (OVA1 was reported to correctly identify 76% of malignancies missed by CA-125) [5]; decreased specificity and positive predictive value were reported, resulting in increased benign tumor referral rates [5,6]. Clearly, there is an opportunity for additional serum biomarkers to further increase the sensitivity and/or specificity with which EOC is diagnosed and in so doing, impact significantly on mortality due to this malignancy.

MicroRNAs (miRNAs) are a class of small (17-22 nucleotide) non-coding RNA molecules that negatively regulate gene expression. They display distinct expression profiles in tumors and are able to differentiate between cancer and normal tissue, as well as histological subtypes of ovarian cancer [7-10]. A study in breast cancer reported a correlation between miRNA expression in tissue and sera [11]. Circulating miRNAs are stable after multiple freeze/thaw cycles, RNase A treatment and incubation at room temperature, with several studies investigating these as markers of cancer [12-15].

We hypothesized that miRNA over-expressed in SEOC cell lines may be found in abundance in serum from women with SEOC compared to age-matched healthy women. Furthermore, we proposed that miRNAs that are invariant between SEOC and healthy cohorts may have utility as endogenous controls to normalize serum miRNA levels in quantitative PCR (qRT-PCR) analyses.

## Methods

### Cell lines

The human SEOC cell lines OVCAR-3 [16], PE01 [17], OV167, OV202 and normal human ovarian surface epithelial (OSE(tsT)) cells [18,19] were used in this study. The sources of these cells, culture conditions and authentication by short tandem repeat profiling (The Lady Fairfax CellBank Australia, Westmead, New South Wales, Australia) are detailed in Additional file 1: Table S1. All experiments were performed using cells within 5 passages of those selected for authentication.

### RNA Extraction from cell lines

RNA for miRNA microarrays was extracted using Trizol (Invitrogen, Life Technologies Australia Pty Ltd, Mulgrave VIC, Australia) and for qRT-PCR using the mirVana PARIS kit (Ambion, Applied Biosystems, Foster City, CA, USA), according to each manufacturers’ protocol.

### miRNA expression profiling

miRNA expression profiling was performed using 2-color (Cy-3 *versus* Cy-5) Exiqon MiRCURY^TM^ Locked Nucleic Acid arrays (Geneworks Pty Ltd., Thebarton, SA, Australia; miRBase 8.2 for human; Adelaide Microarray Centre, SA, Australia). RNA from the cell lines was compared to a commercial RNA pool (Ambion FirstChoice Human Total RNA Survey Panel, Applied Biosystems). Triplicate and duplicate arrays were performed on the OSE(tsT) and SEOC cell lines respectively, and included a dye-swapped array for each cell line.

For each array, background correction was performed by fitting a mixture model of a normal and exponential distribution where normal distribution captures the non-expressed probes and exponential distribution the expressed probes [20]. Loess print-tip normalization was performed within arrays. Scale normalization was performed between arrays. As each probe was printed as adjacent duplicate spots, these were expected to be positively correlated. Therefore, technical replicates were corrected for using the duplicateCorrelation function to assess differential expression via Linear Models for Microarray data (LIMMA), using the value for the average correlation to merge data from duplicate spots [21,22].

The raw microarray data have been deposited in NCBI's Gene Expression Omnibus [23] and are accessible through GEO Series accession number GSE35951 (http://www.ncbi.nlm.nih.gov/geo/query/acc.cgi?acc=GSE35951). GeneSpring GX (v11.0.1, Agilent Technologies) was used for analysis.

### Specimen cohorts and serum collection

Serum from patients with SEOC (*n* = 28) and age-matched healthy female volunteers (*n* = 28) were obtained from the Kolling Institute of Medical Research Gynecological Tumor Bank (Human ethics protocol #0812-259 M). Healthy volunteers had no personal or family history of cancer and had not been hospitalized in the 6 months prior to collection. The majority of cancer patients (70%; 20/28) were diagnosed with the International Federation of Gynecology and Obstetrics (FIGO) stage 3B/C SEOC; other clinical data are summarized in Table 1.

**Table 1:** Clinical variables of serous ovarian cancer patients in the study

		**Patient**	**Healthy volunteers**
		**(*n* = 28)**	**(*n* = 28)**
***Age (years)***			
Median		63 ± 11	64 ± 12
Minimum		30	30
Maximum		77	79
***Histological Grade***			
Moderately differentiated		11	
Poorly differentiated		11	
Well-differentiated		1	
Unknown		5	
***Site***			
Tubal		3	
Peritoneal		4	
Ovary		20	
Endometrial		1	
***FIGO Stage***			
4		6	
4B		1	
3C		17	
3B		3	
2C		1	
***CA-125 Result***			
Yes		19	
No		9	
***BRCA1/2 mutation***			
Yes		3	
Unknown		24	
No		1	
***Post Menopausal***			
Yes		23	
No		2	
Unknown		2	
***Pre-operative Chemotherapy***			
Yes		9	
No		19	
***Prior cancer***			
Yes		2 (breast, Craniopharyngioma)	
No		26	
***Tumour Size***			
mean		59.3 ± 33.6	
min		20	
max		140	
***Degree of cytoreduction***			
< 2 cm		8	
> 2 cm		8	
No Macroscopic		13	
Unknown		1	
***Survival Statistics***			
Dead of disease		12	
Recurred		12	
No evidence of disease		4	

### Total RNA extraction from serum

Total RNA was extracted from 500 μL serum using the miRVANA PARIS kit according to the manufacturer’s protocol, except that the acid-chloroform precipitation step was performed twice[12] and samples were eluted twice from the column with 105 μL H_2_0 pre-heated to 95°C.

### qRT-PCR quantification of miRNA expression in serum and cell lines

miRNA were quantified by TaqMan miRNA assays (Applied Biosystems) following reverse transcription (TaqMan MicroRNA RT Kit, Applied Biosystems) of 10 ng (cell line) or 40 ng (serum) RNA. To reduce the possible high intra-assay variance introduced by low abundant miRNA, a pre-amplification step using TaqMan PreAmp Master Mix (Applied Biosystems) was performed for all serum RNA samples with cycling conditions: 2 min at 50°C, 10 min at 95°C followed by 12 rounds of 15 sec at 95°C and 1 min at 60°C. The resulting pre-amplification products were diluted 1:2.3 and 5 μL used for each 10 μL miRNA assay. Cycle threshold (C_T_) values above the determinable range (up to 45) were assigned a C_T_ of 45. All reactions were performed in triplicate and initially normalized to a calibrator (pool of serum and cell lines RNA) that was included in every plate.

Small nucleolar RNA C/D box 7 (SNORD7, also referred to as Z30) was chosen as the endogenous normalizer for cell line miRNA expression because of its low variance and moderate abundance across the NCI-60 cell lines [24]. However, it was not suitable for normalization of serum miRNA as the levels in serum were below the limit of detection of our assay (data not shown). As there are no endogenous miRNA established as normalizers for serum miRNA from women with or without SEOC, we assessed a number of candidates and compared these with serum volume normalization, the latter currently the most common strategy for normalization of serum miRNA [25,26].

### Statistical analysis

The power of selected miRNA to distinguish between cancer and healthy patient groups was assessed by receiver operating characteristic (ROC) area under the curves (AUC) analyses using SPSS software v20 (SPSS Australasia Pty Ltd., Chatswood, NSW, Australia). Leave one out cross validation was also performed using SPSS. Statistical significance was determined by the Mann–Whitney *U* Test (non-parametric data) or one-way ANOVA (LSD Post-hoc test). A *P*-value of ≤ 0.05 was regarded as significant. Linear regression analysis was performed to assess possible correlations between expression of miRNAs and clinical characteristics using Microsoft Office Excel 2007.

## Results

### miRNA differentially expressed between SEOC and OSE(tsT) cell lines

**Figure 1:**
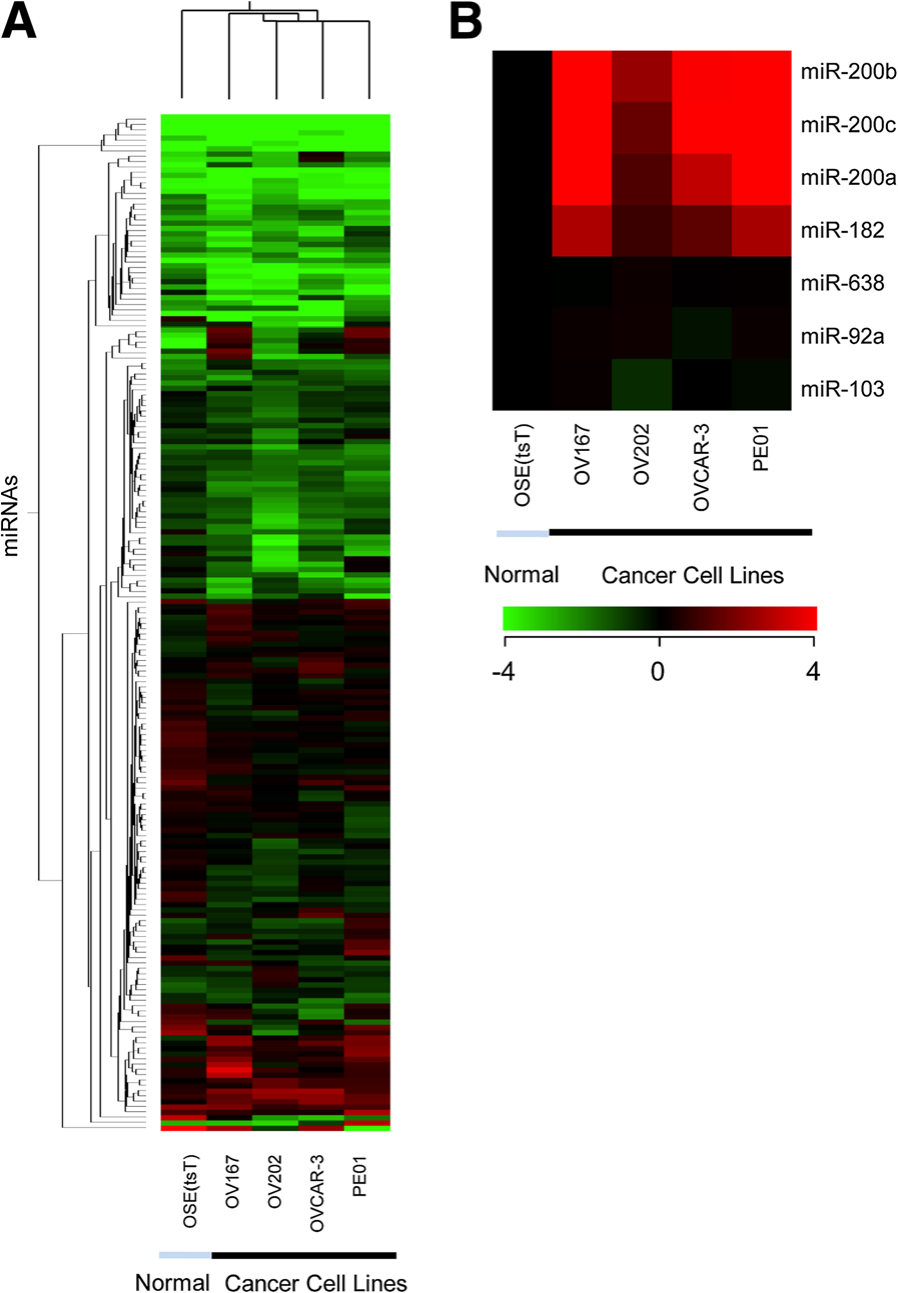
**miRNA microarray profiling of SEOC and OSE(tsT) cell lines.** [**A**] Heatmap of supervised hierarchical clustering (Euclidean, Centroid) of miRNAs with at least a 2-fold difference in one or more SEOC cell lines relative to OSE(tsT) cells; miRNA are listed in Additional file 1: Table S2. [**B**] Heatmap showing expression relative to OSE(tsT) cells (ie OSE(tsT) set to log_2_0) for candidate biomarkers in SEOC cell lines (miR-200a, b, c, and miR-182) and candidate endogenous controls for normalization (miR-638, miR-92a and miR-103). Heatmaps were generated using Genespring software.

**Figure 2:**
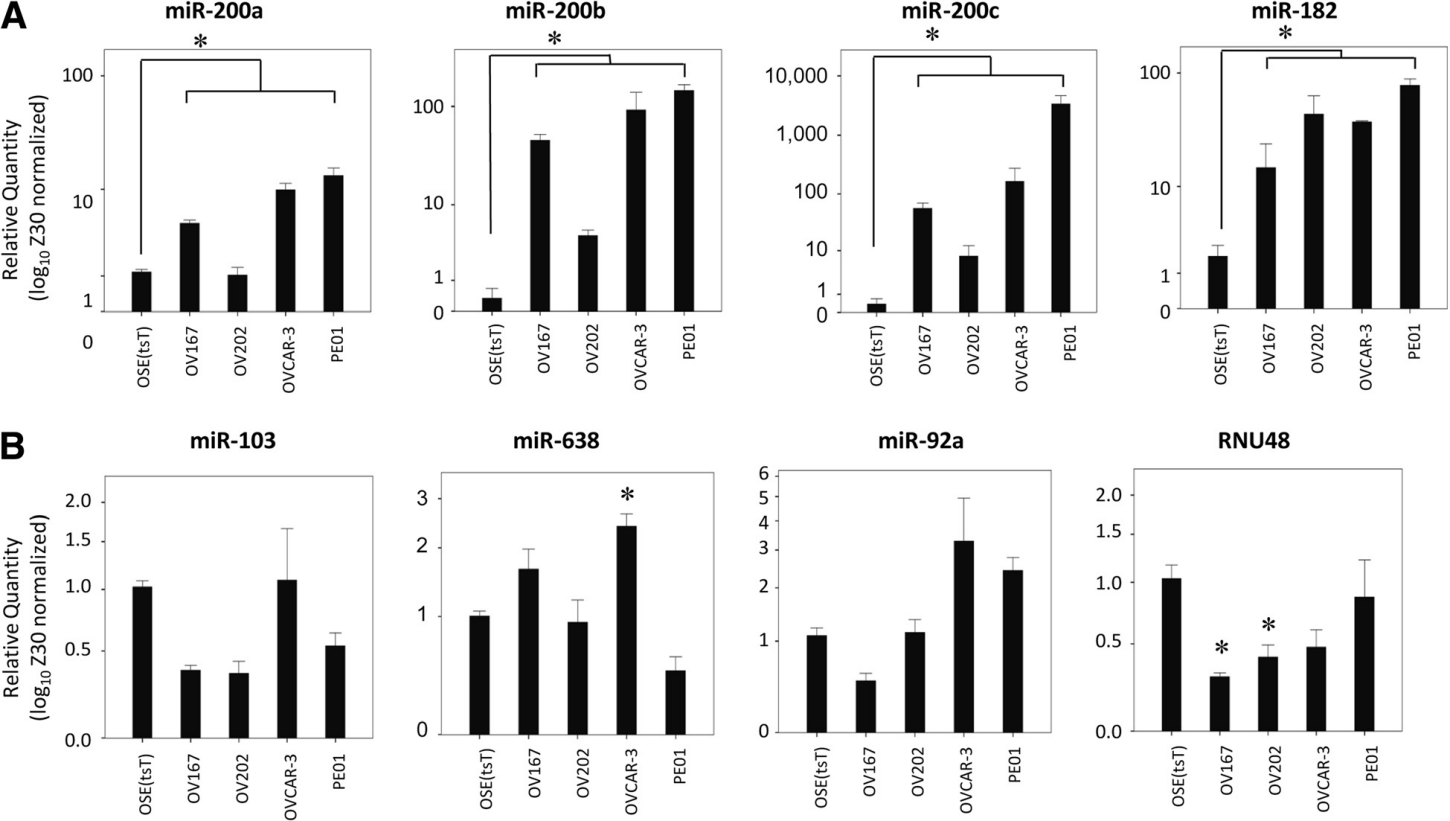
**Cell line expression of miRNA for investigation.** [**A**] The expression of candidate biomarkers, miR-200a, miR-200b, miR-200c and miR-182 and [**B**] the expression of candidate normalizers, miR-103, miR-638, miR-92a and RNU48 in SEOC and OSE(tsT) cell lines by qRT-PCR normalized to Z30 and plotted as log_10_ ratios. Results are shown for 3 independent experiments with each experiment performed in triplicate. *P < 0.05, one-way ANOVA (LSD post-hoc TEST).

To identify differentially expressed miRNAs in SEOC cell lines, miRNA expression profiling was performed. Supervised hierarchical clustering clearly separated the SEOC from OSE(tsT) cell lines (Figure 1A, Additional file 1: Table S2). Candidate biomarkers miR-182, miR-200a, b and c were selected based on > 2-fold (log_2_) over-expression in at least 3 of the 4 SEOC cell lines relative to OSE(tsT) cells (Figure 1B) and confirmed by qRT-PCR (*P* < 0.05, 1-way ANOVA; Figure 2A).

### miRNA investigated for uniform expression between SEOC and OSE(tsT) cell lines

In order to select miRNAs with potential suitability as endogenous controls for normalization, we generated a list of 451 miRNAs that were expressed at a constant level across all SEOC cell lines and OSE(tsT) (test based on one-way ANOVA with asymptotic *P* values > 0.05 using Benjamini and Hochberg False Discovery Rate for multiple testing correction). From this list, miRNA previously reported to be detected in serum or plasma were selected for further analysis, specifically, miR-92a, miR-103 [12,14,27] and miR-638 [28] (Figure 1B). miR-103 had also been reported to have similar expression across ovarian cancer and normal tissues [29]. Small nucleolar RNA, C/D box 48 (RNU48) was also assessed as it was used as a normalizer for miRNA studies in solid tumours [30] and known to be expressed in serum [12].

qRT-PCR assays were performed on RNA from the cell lines and confirmed that miR-92a and miR-103 were not differentially expressed in any of the SEOC cell lines compared to OSE(tsT) cells (*P* > 0.05; Figure 2B). However, miR-638 was overexpressed in OVCAR-3 cells, and RNU48 underexpressed in OV167 and OV202 cells relative to OSE(tsT) cells (*P* < 0.05, ANOVA LSD post-hoc test; Figure 2B) suggesting that miR-638 and RNU48 may not be appropriate to use as normalizers.

### Levels of candidate endogenous miRNA normalizers in serum

**Figure 3:**
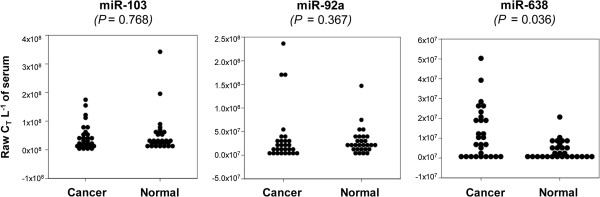
**Candidate miRNA endogenous control levels in serum adjusted for volume (ΔCT/L of serum assayed) for SEOC patients (*n* = 28) and healthy controls (*n* = 28).** P-values determined by the Mann–Whitney U Test.

The levels of each candidate normalizer (miR-103, miR-92a, miR-638 and RNU48) were assessed in serum RNA from the SEOC (*n* = 28) and age-matched healthy donor (*n* = 28) cohorts by qRT-PCR assays following a pre-amplification step. Despite pre-amplification, RNU48 failed to amplify in 36% (20/56) of serum samples and was eliminated as a candidate normalizer. The remaining results were assessed following normalization to volume of serum used for reverse transcription (Figure 3) [26].

No correlations were found between subject age and miR-103, miR-92a or miR-638 levels (Additional file 1: Figure S1), and no significant differences between the SEOC and healthy groups were observed for miR-103 (*P* = 0.768) or miR-92a *(P* = 0.367; Figure 3). However, for miR-638, a trend towards higher C_T_s in the SEOC cohort was observed (*P* = 0.063; Figure 3). This finding, in conjunction with the significant differential expression observed in a single SEOC cell line (*P* < 0.05, Figure 2B), led to our rejection of miR-638 as a candidate normalizer. miR-92a was also rejected as it had recently been reported to be differentially expressed in serum of women with EOC [27]. From these results, miR-103 was chosen as the endogenous normalizer for serum miRNA.

### Candidate biomarker miRNA expression in serum

We next assessed the miRNA selected as candidate biomarkers (miR-200a, b, c and miR-182) in serum RNA from the SEOC and healthy cohorts. Volume adjusted C_T_s were normalized to miR-103.

**Figure 4:**
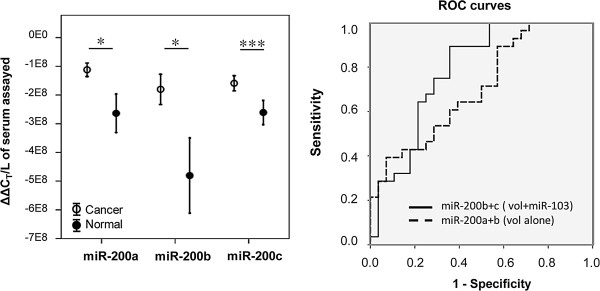
**miR-200a, b and c are significantly elevated and predictors of ovarian cancer in patient serum.** [**A**] Mean expression of miR-182, miR-200a, miR-200b and miR-200c in cancer and normal serum; volume adjusted values normalized to miR-103 (ΔΔC_T_/L^-1^ of serum assayed) +/- SEM (*N* = 56). *P < 0.05, ***P < 0.0005, Mann–Whitney *U* Test [**B**] ROC curves for miRNA combinations with the highest AUC for volume adjusted results (vol alone; AUC = 0.705) and volume adjusted results normalized to miR-103 (vol + miR-103; AUC = 0.784).

The levels of miR-200a, b and c were significantly elevated in the SEOC cohort relative to the healthy group (Figure 4A), with miR-200c the most significantly different between the 2 groups (*P* < 0.0005). For miR-182 the levels between the 2 cohorts did not reach significance (data not shown). No correlations between the 4 miRNA and subject age were found (Additional file 1: Figure S1).

### Correlation of serum miRNA levels with clinical characteristics

To determine the diagnostic potential of selected miRNAs, ROC-AUC curves were constructed for miR-200a, b and c. A multivariate model that combined miR-200b + c with normalization by serum volume and miR-103 gave the highest ROC-AUC for discriminating women with SEOC from healthy women (AUC = 0.784; Table 2, Figure 4B). In the absence of a separate validation cohort, leave one out cross validation was performed for this model (miR-200b + c) and gave an identical AUC value. For normalization by serum volume alone, the strongest model combined miR-200a + b (AUC = 0.705; Table 2, Figure 4B).

**Table 2:** ROC-AUC analysis for miR-200a, b and c

**miRNA**	**AUC**	***P *value**	**Sensitivity (%)**	**Specificity (%)**
***Normalized by volume and miR-103***				
miR-200a	0.675	0.025	85.7	35.7
miR-200b	0.722	0.004	85.7	35.7
miR-200c	0.727	0.004	71.4	57.1
**miR-200b + c**	**0.784**	**<0.001**	**78.6**	**46.4**
***Normalized by volume alone***				
miR-200a	0.648	0.057	82.1	35.7
miR-200b	0.684	0.018	89.3	32.1
miR-200c	0.702	0.010	75	53.6
**miR-200a + b**	**0.705**	**0.008**	**85.7**	**42.9**

Pre-operative CA-125 values were recorded where available (Table 1) and compared with miRNA expression in serum. No associations between any of the 3 miRNAs (miR-200a, b or c) and CA-125 levels were found (Additional file 1: Table S3). A previous small study found that sera from 3 patients with low CA-125 levels had elevated miR-92a levels [27]. Therefore, we compared CA-125 levels with miR-92a expression. No association was observed (Additional file 1: Table S3). No significant associations were found between the expression of any of the miRNA investigated and tumor size, progression free intervals, survival (Additional file 1: Table S3) or neoadjuvant therapy (data not shown.)

## Discussion

We investigated the levels of 4 miRNA (miR-200a, b, c and miR-182) in the serum of patients with SEOC (*n* = 28) and healthy age-matched volunteers (*n* = 28) and identified miR-200c as having the most significantly different levels between the 2 groups (*P* < 0.0005, Mann–Whitney *U* Test). A multivariate model combining miR-200b and miR-200c gave the best predictive power to discriminate SEOC and healthy serum, with a ROC-AUC of 0.784.

In the absence of a second independent set of samples, the predictive performance of miR-200b + miR-200c was tested by leave one out cross validation analysis and resulted in an identical ROC-AUC. While a second set of independent samples may be preferable for validation testing, cross validation testing overcomes the recognized difficulty of obtaining additional cohorts.

A challenge for accurately determining the levels of miRNA in serum by qRT-PCR is the fact that there are currently no endogenous miRNA firmly established for normalization of serum miRNA despite the large number of studies examining serum miRNA as either biomarkers or normalizers [26,31]. Of the three smaller studies that have reported miRNA analysis of serum from patients with ovarian cancer, only one used qRT-PCR and compared serum from patients with ovarian cancer (mixed subtypes) with serum from healthy women (Table 3). This study chose miR-142-3p as the normalizer. However we found this miRNA to be consistently down-regulated in the SEOC cell lines relative to OSE(tsT) (Additional file 1: Table S2). For the current study, miR-103, miR-92a, miR-638 and RNU48 were assessed as potential endogenous normalizers and miR-103 chosen as it had low differential expression between the SEOC and healthy cohorts (*P* = 0.768). miR-103 has previously been found to be highly invariant across a number of cancerous and adjacent normal tissues including ovarian tissue, and ranked in the top 3 of 16 candidates for use as a normalizer for tissue studies [29]. It has also been shown to be present in serum and not differentially expressed between serum samples from healthy controls and gastric cancer patients [26]. Our findings in SEOC patients and an age-matched healthy cohort are in agreement with these previous reports.

**Table 3:** Serum miRNAs in ovarian cancer

**Cohort**	**Subtype**	**Stage**	**RNA origin**	**Platform**	**Result**	**Reference**
2 cancer	unknown	IV	1 mL serum	microarray (custom)	Clustered with other cancers and separate from normals	[32]
9 cancer	Mixed, 60% serous	I-IV	250uL serum	TaqMan qRT-PCR array	21 differentially expressed miRNA including miR-21, 92, 93, 29a, 126	[27]
4 normal		----				
19 cancer	Mixed, 60% serous	I-IV	250uL serum	TaqMan qRT-PCR Assays (normalizer: miR-142-3p)	Increased in cancer *versus* normal: miR-21, 92, 93, 29a, 126	[27]
11 normal		----				
4 cancer	serous	IV	Matched tumor and serum (exosomes from 2.5 mL serum)	microarray (custom)	Similar levels between tumor and serum suggesting a tumor origin for: miR-21, 141, 200a, 200b, 200c, 203, 205	[15]
30 cancer	serous	I (10)	Exosomes from 2.5 mL serum	microarray (custom)	Increased in cancer *versus* benign: miR-21, 141, 200a, 200b, 200c, 203, 205, 214, 215	[15]
		II (10)				
		III (10)				
10 benign	adenoma	----				
28 cancer	Serous	II-IV	500 uL serum	TaqMan qRT-PCR Assays (normalizer: miR-103)	Increased in cancer *versus* normal: miR-200a, 200b, 200c	This study
28 normal	Age-matched	----				

A highly consistent finding from miRNA profiling of primary tumor samples of SEOC is over-expression of miR-200a, b and c (Table 4) [7,8,10]. These 3 miRNA were also the most highly elevated in the SEOC cell lines tested in our study. There is a strong correlation between the levels of these miRNAs in exosomes isolated from matched serum and tumor cultures from patients with Stage III SEOC, providing evidence of tumor origin of these circulating miRNAs (Table 3) [15]. Furthermore, increased levels of these miRNA were not found in women with benign ovarian disease (Table 3). The current study extends this work, showing that miR-200a, b and c, are positive predictive classifiers of SEOC. It demonstrates that standard RNA extraction from serum without the need for the capture and isolation of exosomes, is suitable for assessment of tumor-derived serum miR-200a, b and c in patients with SEOC. In addition, the age range of the 56 subjects (SEOC patients and age matched healthy volunteers) tested in this study spanned almost 5 decades, enabling assessment of subject age as a possible confounder of serum miRNA levels. We found no association between subject age and serum miRNA levels for miR-200a, b, c, (or miR-103, miR-92a, miR-182, miR-638 or RNU48).

**Table 4:** Reports of miR-200 in ovarian cancer

**miRNA**	**Findings**	**Reference**
200a/b/c	High expression in ovarian cancer samples	[7,8,10]
	High expression correlated with decreased progression-free survival	[8]
	Decreased in tumors with high β-tubulin III levels	[33]
200a	Increased in 43% of primary ovarian carcinomas, associated with high-grade and late-stage tumors	[9]
	High expression correlated with recurrence free survival and, or, overall survival	[34,35]
	Increased tumour growth, targets p38α and modulates oxidative stress signature	[34]
	Sensitized tumors to paclitaxel	[34]
200c	Targeted *TUBB3* for degradation leading to increased paclitaxel sensitivity	[36]
	Low levels associated with incomplete response to paclitaxel–carboplatin chemotherapy and recurrence	[33]
	Decreased adhesion to basement membrane complex	[36]
	Repressed ZEB1, decreased migration and invasion in HEY cells	[36]

As stated above, there is evidence that the elevated levels of miR-200a, b and c found in serum from women with SEOC are derived from the cancerous tissue and thus may reflect high expression of the miRNA in SEOC tissue. There is general but not complete concordance that high expression of at least one member of the miR-200 family in SEOC tissue confers improved survival and/or better response to paclitaxel treatment while paradoxically leading to increased proliferation (Table 4) [8,33-36]. Improved survival may be due to repression of the miR-200c target, *ZEB1* a key transcriptional regulator of epithelial to mesenchymal transition, and the maintenance of a less invasive and aggressive epithelial phenotype [36,37]. Improved chemosensitivity may be due to down regulation of another miR-200c target, *TUBB3*/class III beta-tubulin, a component of microtubules that binds to paclitaxel [33,36]. It may also be due to miR-200a driving an oxidative stress response signature thereby increasing sensitivity to paclitaxel induced reactive oxygen species production [34]. With serum miR-200 levels shown to mirror the levels in the SEOC tissue [15], these data suggest that serum miR-200a, b, or c levels may have potential as indicators of prognosis or response to therapy. In the current study, the diverse range of chemotherapy administered to patients precluded correlation of miR-200 family levels and response to therapy.

## Conclusions

We found that miRNA elevated in SEOC cell lines were also elevated in serum from patients with SEOC validating this approach to candidate biomarker selection. A number of these miRNA had previously been reported to be highly expressed in ovarian cancer tissue, providing further evidence that circulating miRNAs are shed from the cancer. We propose that specific miRNAs may have utility as serum biomarkers for SEOC and identified that a small marker panel combining miR-200b and c normalized to serum volume and miR-103, is a positive classifier of SEOC. Testing of a larger cohort that includes patients with early stage disease is warranted to determine whether the addition of miR-200a, b or c, to a panel of serum biomarkers may improve diagnostic sensitivity.

## Abbreviations

SEOC: Serous epithelial ovarian cancer; miRNAs: microRNAs; CA-125: Cancer Antigen 125; qRT-PCR: Quantitative RT-PCR; OSE(tsT): Normal human ovarian surface epithelial cells; FIGO: International Federation of Gynecology and Obstetrics; SNORD7,Z30: Small nucleolar RNA C/D box 7; ROC-AUC: Receiver operating characteristic area under the curves; RNU48: Small nucleolar RNA, C/D box 48; C_T_: Cycle threshold.

## Competing interests

The authors declare that they have no competing interests.

## Authors’ contributions

CWSK and JYH performed the experiments. GBG and JM collected the clinical data. CWSK, MAH, DJM and VMH analyzed the data. CWSK, DJM and VMH wrote the paper. MAH, DJM and VMH designed the study. All the authors read and approved the final manuscript.

## Pre-publication history

The pre-publication history for this paper can be accessed here:

http://www.biomedcentral.com/1471-2407/12/627/prepub

## Supplementary Material

Additional file 1 **Table S1.** Cell lines and cell line typing. **Table S2.** Table of differentially expressed miRNAs with at least 2-fold change (absolute log_2_ fold change) in at least 1 of 4 cell lines relative to OSE(tsT). **Figure S1.** Serum miRNA levels relative to age. **Table S3.** Pairwise correlation values for serum miRNA levels and CA-125, tumor size, progression free interval or overall survival. Click here for file
