# Supplementary material for: Elevated levels of circulating microRNA-200 family members correlate with serous epithelial ovarian cancer
Source: BMC Cancer. 2012 Dec 28;12:627. doi: 10.1186/1471-2407-12-627 (PMC3542279; doi:10.1186/1471-2407-12-627)
Supplement: Additional file 1 — Table S1. Cell lines and cell line typing. Table S2. Table of differentially expressed miRNAs with at least 2-fold change (absolute log2 fold change) in at least 1 of 4 cell lines relative to OSE(tsT). Figure S1. Serum miRNA levels relative to age. Table S3. Pairwise correlation values for serum miRNA levels and CA-125, tumor size, progression free interval or overall survival. [file 1471-2407-12-627-S1.pdf]

Table S1. Cell lines and cell line typing

The human SEOC cell line OVCAR-3 was purchased from the American Tissue Culture Collection (Manassas, VA, USA) The PE01 cell line was derived from a poorly differentiated ovarian adenocarcinoma and obtained from Dr. S.P Langdon (Cancer Research UK Centre, University of Edinburgh, Edinburgh, United Kingdom). SEOC cell lines OV167 and OV202; and normal human ovarian surface epithelial (OSE(tsT)) cells were kindly provided by Drs. C. Conover and K. Kalli (Mayo Center, Rochester, MN, USA).

All SEOC cell lines were cultured in RPMI 1640 (Gibco, Life Technologies Australia Pty Ltd, Mulgrave, VIC, Australia) supplemented with 10% (volume/volume) fetal bovine serum (SAFC Bioscience, Brooklyn, VIC, Australia) at 37°C with 5% CO2. Cells were grown to 80% confluence prior to harvesting for RNA extraction. The HOSE cell line expresses a temperature sensitive mutant of the SV40 large T-antigen, which enables it to proliferate at 34°C but is inactivated at 39°C. OSE(tsT) was cultured in MCDB 105:Medium 199 (1:1) (Sigma-Aldrich Corporation, Sydney, NSW, Australia) and supplemented with 15% fetal bovine serum (SAFC Bioscience) at 34°C in 5% CO2. After plating, cells were allowed to attach for 24 hours at 34°C before incubating at 39°C in 5% CO2 for 72 hours prior to RNA extraction.

Cell typing was performed by The Lady Fairfax CellBank Australia (Westmead NSW 2145, Australia) using the AmpFl STR Identifiler PCR Amplification Kit (Applied Biosystems), a 16 loci (15 STR loci plus Amelogenin) STR multiplex kit. OVCAR-3 was correctly identified. To our knowledge, profiles for OSE(tsT), OV167, OV202 and PE01 have not previously been available.

**No.**	1	2	3	4
**Sample Name**	OSE(tsT)	OV167	OV202	PE01
**Sample ID**	10-375	10-376	10-377	10-378
**D8S1179**	11,16	10,12	8,11	13,14
**D21S11**	28,29	30.2	28,32.2	32.2
**D7S820**	10	9,10	8,9	10
**CSF1PO**	10,12	12	9,10	10,12
**D3S1358**	17,18	16	17,18	16
**TH01**	6,7	8	9,9.3	9.3
**D13S317**	11,15	12	11	10
**D16S539**	9,13	10	9,11	9
**D2S1338**	18,23	17	17,18	20,21
**D19S433**	15	12,15	13	13,15
**vWA**	14,18	15,18	16,18	15,16
**TPOX**	8,12	9	8,9	9,11
**D18S51**	14,18	19	12	16,17
**Amel**	X	X	X	X
**D5S818**	11,12	11	11,13	11,12
**FGA**	21,23	21	21,22	20

Table S2. Table of differentially expressed miRNAs with at least 2-fold change (absolute $\log_2$ fold change) in at least 1 of 4 cell lines relative to OSE(tsT). miRNAs assessed as serum biomarkers are highlighted in yellow.

	[OV167] vs [OSE(tsT)]		[OV202] vs [OSE(tsT)]		[OVCAR3] vs [OSE(tsT)]		[PE01] vs [OSE(tsT)]	
Name	Absolute Fold Change	Regulation	Absolute Fold Change	Regulation	Absolute Fold Change	Regulation	Absolute Fold Change	Regulation
hsa-miR-1425p	1.9487	down	2.7654	up	1.4464	down	1.0886	up
hsa-miR-124a	2.7289	up	1.0377	up	2.5432	down	3.5044	down
hsa-miR-522	3.0755	up	1.2687	up	12.3877	up	2.3027	up
hsa-miR-215	2.0922	down	1.5610	down	2.0000	down	1.1368	down
hsa-miR-143	1.5911	down	12.9287	up	1.1427	down	1.6906	down
hsa-miR-199a*	6.6962	down	1.2842	down	1.2976	down	3.4422	down
hsa-miR-521	1.1540	up	1.6208	up	18.7221	up	3.4204	up
hsa-miR-126	9.4370	down	1.4431	up	2.5521	up	2.1226	down
hsa-miR-141	20.1587	up	1.3787	up	2.9862	up	21.6431	up
hsa-miR-223	2.2166	down	1.2577	down	1.6142	down	1.3835	down
**hsa-miR-200b**	**36.8221**	**up**	**5.1874**	**up**	**15.4818**	**up**	**28.2465**	**up**
hsa-miR-133a133b	3.3694	down	1.8596	up	1.0792	up	1.1996	down
hsa-miR-422b	10.6726	up	3.0755	up	7.3022	up	3.4422	up
hsa-miR-5123p	4.1101	down	2.0837	down	1.5984	down	3.0720	down
**hsa-miR-200a**	**28.9736**	**up**	**2.3443**	**up**	**8.2344**	**up**	**36.4202**	**up**
hsa-miR-26a	1.2219	down	1.5201	down	1.1264	down	5.7292	up
hsa-miR-451	2.1798	down	1.1796	up	1.4085	down	2.5832	down
hsa-miR-663	1.2058	up	3.4343	up	3.7646	up	4.6107	up
hsa-miR-519b	2.8563	down	1.0222	down	1.5351	up	3.1311	down
hsa-miR-195	2.6421	down	1.7211	up	1.1837	up	1.1375	up
hsa-miR-363	2.7116	up	1.2177	up	1.1600	up	1.1540	up
hsa-miR-637	1.2812	down	1.4641	up	2.4666	up	1.0570	down
hsa-miR-122a	1.0070	up	1.5883	up	2.8679	down	2.1697	down
hsa-miR-519a	4.5237	down	1.8119	down	3.9109	up	1.1348	up
hsa-miR-205	1.1500	down	1.4914	down	3.6322	up	51.9242	up
hsa-miR-519e	1.0443	down	2.2934	up	1.4515	up	1.0792	up
**hsa-miR-200c**	**26.3549**	**up**	**3.0738**	**up**	**21.7433**	**up**	**30.0647**	**up**
hsa-miR-30b	1.0736	down	1.1567	up	1.3779	down	4.2281	up
hsa-miR-548c	4.5525	down	2.7590	down	1.6349	down	1.1205	down
hsa-miR-1423p	17.0890	down	5.5790	down	2.3743	down	3.0157	down
hsa-miR-30d	1.6254	down	1.5902	up	1.1722	up	2.5476	up
hsa-miR-20b	2.2671	up	1.0163	up	1.0528	down	1.7004	up
hsa-miR-30a5p	15.2334	up	1.5219	down	1.2276	down	3.1858	up
hsa-miR-3693p	2.3497	up	1.3660	up	1.4464	up	2.2076	up
hsa-miR-26b	2.4033	down	2.7179	down	1.8564	down	1.3660	down
hsa-miR-9	4.0255	down	6.9604	down	2.1019	down	3.7235	down
hsa-miR-30e3p	1.7787	up	1.4573	up	2.6867	up	2.1597	up
hsa-miR-34c	1.9130	up	1.8192	up	4.2110	down	1.1375	up
hsa-let-7c	3.2735	down	1.6198	down	1.5520	up	2.0444	up
hsa-miR-140	4.7596	down	1.2534	down	1.7787	down	2.4340	down
hsa-miR-146b	1.7401	down	2.4228	down	3.0088	down	1.0638	down
hsa-miR-145	1.1996	down	2.3094	up	1.7994	down	1.5131	up
hsa-miR-603	1.4061	down	2.1672	up	1.0128	up	1.1166	down
hsa-miR-30c	3.4903	up	1.3645	up	1.1755	up	1.1179	up
hsa-miR-5153p	9.4479	down	2.0209	down	9.0317	down	12.5316	down
hsa-miR-455	1.3165	down	2.2064	down	1.6096	down	2.5213	down
hsa-miR-429	7.1934	up	2.4214	down	1.6925	up	3.3154	up
hsa-miR-199b	1.1335	down	5.9107	down	1.8067	down	2.5997	down
hsa-miR-1	5.5758	down	2.5476	down	1.0631	up	2.9983	down
hsa-miR-449b	1.2212	down	2.3147	down	1.5360	up	1.3387	down
hsa-miR-539	2.5228	down	2.5491	down	1.4340	down	1.5692	down
hsa-miR-10b	3.7886	down	1.4726	up	1.6634	down	3.1914	down
hsa-let-7b	5.8869	down	1.1647	down	3.2154	down	2.5535	up

hsa-miR-29c	1.6692	down	4.7541	down	8.4220	down	1.1362	down
hsa-miR-138	2.4214	down	1.1926	up	1.2390	up	1.0743	down
hsa-miR-197	3.0018	up	2.1116	up	1.4854	up	2.5242	up
hsa-miR-208	1.2184	up	1.6876	down	1.3708	down	2.0069	down
hsa-miR-519c	1.5342	down	1.1851	down	2.2697	down	1.0849	down
hsa-miR-20a	1.7705	up	1.0946	up	1.0622	down	4.3633	up
hsa-miR-671	1.2447	up	2.1006	up	2.1899	up	1.4126	up
hsa-miR-449	1.8067	down	2.4298	down	1.4077	down	1.5701	down
hsa-miR-199a	6.0175	down	6.9483	down	4.6321	down	4.0465	down
hsa-miR-551b	1.6405	down	1.6339	up	1.2534	up	3.7343	up
hsa-miR-5165p	1.5701	up	3.7278	up	1.4624	up	1.5113	up
hsa_SNORD118	2.4144	up	1.3142	up	1.3867	up	1.6320	up
hsa-miR-526b	1.2290	up	3.4046	up	2.0634	up	1.1933	up
hsa-miR-651	1.3021	down	1.4423	down	1.2886	down	2.2397	down
hsa-miR-518e	1.0736	down	2.5982	down	1.2548	down	1.1427	down
hsa-miR-484	1.4666	up	1.7715	up	1.6702	up	2.0669	up
hsa-miR-335	1.5351	down	1.8478	down	2.6087	down	1.6254	down
**hsa-miR-182**	**6.6461**	**up**	**1.8823**	**up**	**2.7416**	**up**	**6.2550**	**up**
hsa-miR-338	3.7646	down	3.9862	down	1.5052	down	2.4453	down
hsa-miR-24	1.0693	down	4.9818	down	2.0023	down	1.3597	up
hsa-miR-509	1.8780	down	1.7552	down	1.1381	down	3.2359	down
hsa-miR-125a	3.0455	down	1.0041	up	1.8171	down	2.8962	down
hsa-miR-95	3.7974	down	3.1492	down	1.9827	down	3.1221	down
hsa-miR-99a	2.1597	down	1.1160	down	1.9611	up	4.8010	down
hsa-miR-30e5p	1.1342	down	1.5929	down	2.1987	down	1.5601	down
hsa-miR-96	5.1367	up	1.6740	up	3.1675	up	3.8194	up
hsa-miR-175p106a	1.5166	up	1.1681	down	1.0163	up	3.5329	up
hsa-miR-152	2.1949	down	6.6422	down	1.8943	down	1.9408	down
hsa-miR-135b	7.3700	up	1.1083	down	1.5387	up	2.2410	up
hsa-miR-379	1.2541	down	3.1969	down	1.4456	down	1.4012	down
hsa-miR-194	2.1873	down	2.6512	down	1.8812	down	1.7674	down
hsa-miR-410	2.3770	down	1.9577	down	2.0729	down	2.8366	down
hsa-miR-16	1.3621	up	2.1798	down	1.2687	up	1.7849	up
hsa-miR-18b	1.9086	up	1.5210	up	1.5637	up	3.9655	up
hsa-miR-578	2.2921	down	1.5280	down	1.7828	down	1.4232	down
hsa-miR-98	1.1920	down	2.4340	down	1.2886	down	1.1225	up
hsa-miR-18a	3.3772	up	1.7482	up	1.5619	up	5.8801	up
hsa-miR-376a	6.1974	down	5.5854	down	8.5841	down	14.0907	down
hsa-miR-29a	1.1634	up	6.5508	down	22.6536	down	1.2404	up
hsa-let-7d	1.2233	down	3.2622	down	1.5351	down	1.1494	down
hsa-miR-33	1.2135	up	1.2753	down	2.2671	down	1.1776	down
hsa-let-7g	1.2827	down	3.2024	down	1.6519	down	1.4431	down
hsa-miR-99b	1.7013	down	1.0540	up	1.1961	up	2.1911	down
hsa-miR-514	5.4076	down	3.9724	down	3.3346	down	1.9285	down
hsa-miR-525*524	1.9397	down	2.3308	down	1.2233	down	1.1460	up
hsa-miR-518f	1.6896	down	2.2842	down	1.2519	down	1.0975	down
hsa-miR-218	1.3203	up	1.2665	up	1.2128	up	2.5068	up
hsa-miR-653	2.1861	down	1.4298	down	1.0780	down	1.0081	up
hsa-miR-384	2.0562	down	2.0035	down	1.6358	down	2.2076	down
hsa-miR-4255p	1.2368	up	1.5219	down	2.0058	up	1.0509	up
hsa-miR-9*	1.1045	down	7.3785	down	1.3249	down	1.8932	down
hsa-miR-626	1.4557	up	1.4582	up	2.5388	up	1.1940	down
hsa-miR-23a	1.1057	down	9.0945	down	1.9053	down	1.6386	up
hsa-miR-27a	1.1540	down	2.8268	down	1.1303	down	2.1672	up
hsa-miR-15b	1.5035	up	1.4632	down	2.3147	up	1.3457	up
hsa-miR-200a*	1.0780	up	1.3187	down	2.4780	down	1.1109	down
hsa-miR-411	1.5404	down	1.8769	down	1.2842	down	2.4467	down
hsa-miR-25	2.0729	down	2.1386	down	1.3939	down	1.4557	down
hsa-miR-22	2.3743	down	2.2038	down	9.1419	down	2.6759	down
hsa_SNORD6	2.7992	up	1.1427	up	1.0192	up	1.8213	up
hsa-miR-148b	1.0668	up	1.3134	up	2.7195	up	1.0674	down

hsa-miR-526b*	1.7201	down	3.0262	down	1.5746	down	1.2592	down
hsa-miR-591	3.1329	down	2.8629	down	2.3014	down	3.3173	down
hsa-miR-496	1.3891	down	1.0269	up	1.3210	down	2.3936	down
hsa-miR-182*	1.3496	down	1.6673	down	1.7171	down	2.9949	up
hsa-miR-567	1.9600	up	1.3203	up	2.0397	up	1.0521	up
hsa-miR-557	1.0041	down	1.7391	down	1.7756	down	2.1226	down
hsa-miR-29b	1.8921	down	3.8571	down	2.3134	down	1.2702	up
hsa-let-7i	2.4694	down	8.6438	down	6.2297	down	1.3883	up
hsa-miR-106b	2.0527	up	1.4692	down	1.2724	down	2.0314	up
hsa-miR-518d	1.4666	down	1.7321	down	2.1697	down	1.6558	down
hsa-miR-19a	1.2469	down	1.7092	down	1.1375	down	2.3120	up
hsa-miR-325	1.7112	down	1.6964	down	2.3295	down	2.0634	down
hsa-miR-569	1.4473	down	1.9839	down	2.1522	down	1.1089	up
hsa-miR-553	2.6223	down	2.1411	down	1.7181	down	1.6396	down
hsa-miR-576	2.3525	down	1.4061	down	1.3418	down	1.3629	down
hsa-miR-376b	2.0373	down	8.1775	down	1.5175	down	6.1653	down
hsa-miR-193b	1.5467	down	1.0093	up	1.0786	down	3.9267	up
hsa-miR-137	2.2359	down	1.0558	down	1.2909	down	1.4828	down
hsa-miR-92b	1.4862	down	1.3716	up	1.3295	up	2.0837	down
hsa_SNORD14B	1.8704	up	2.8596	up	1.9031	up	1.8671	up
hsa-miR-487b	1.3716	down	1.2961	up	2.5418	down	1.8161	down
hsa-miR-301	8.1023	up	1.2886	up	1.4012	down	1.1837	up
hsa-miR-125b	9.0005	down	1.6189	down	2.4837	down	3.1711	down
hsa-miR-645	2.3647	down	1.2347	down	1.1989	down	1.0930	up
hsa-miR-614	1.7736	up	2.0765	up	1.0329	up	1.0749	up
hsa-miR-136	1.4398	down	2.0898	down	1.0564	up	1.3550	down
hsa-miR-374	1.1335	up	2.2256	down	1.3931	up	1.7072	down
hsa-miR-330	1.1096	up	2.5758	up	1.1547	down	1.1115	up
hsa-miR-656	1.3036	down	1.4439	down	1.2991	down	2.4880	down
hsa-miR-2993p	1.1507	up	1.4241	down	3.0053	down	3.4641	down
hsa-miR-616	2.8399	up	2.0397	up	1.1920	up	1.5219	up
hsa-miR-4935p	1.0012	up	1.6003	down	2.0012	down	1.9942	down
hsa-let-7a	3.2546	down	7.5292	down	4.7568	down	1.0407	up
hsa-miR-4253p	1.3496	down	1.7471	down	1.4489	down	2.7226	down
hsa_SNORD13	2.5418	up	3.4244	up	3.7149	up	1.3134	up
hsa-miR-607	2.3134	up	1.1290	up	1.5583	up	1.2397	up
hsa-miR-605	1.6837	down	2.0729	down	1.1166	down	1.0222	down
hsa-miR-34a	4.1030	down	14.8168	down	3.5288	down	11.0553	down
hsa-miR-431	2.9299	down	4.7678	down	3.0862	down	3.4903	down
hsa-miR-339	1.4607	down	2.2566	down	1.9241	down	1.6984	down
hsa-miR-100	35.4447	down	13.3152	down	12.7286	down	3.4702	down
hsa-let-7f	1.5538	down	2.0221	down	1.6510	down	1.9431	down
hsa-miR-368	2.0669	down	1.8308	down	1.1076	up	1.5828	down
hsa-miR-5155p	9.3125	down	7.6564	down	2.7023	down	6.0384	down
hsa-miR-3805p	3.4641	down	2.8235	down	1.3875	down	2.6117	down
hsa-miR-130a	4.7541	up	1.1006	down	1.1121	down	1.1968	up
hsa-miR-520d*	2.0813	down	1.8150	down	1.1487	down	1.3827	down
hsa-miR-383	1.1892	down	2.2307	down	1.7053	down	1.3149	down
hsa-miR-184	1.3732	up	1.7623	up	2.1031	up	1.6994	up
hsa-miR-432	1.4794	down	2.2307	down	2.8186	down	1.7994	down
hsa-miR-583	1.0718	down	2.1140	up	1.4167	up	1.4489	up
hsa-miR-639	1.3511	down	2.1873	down	1.4992	down	1.8203	down
hsa-miR-582	1.7880	down	1.9600	down	2.1043	down	2.8008	down
hsa-miR-452*	1.0455	up	2.4425	down	1.3226	down	1.1032	up
hsa-miR-127	1.3028	down	2.3812	down	2.8121	down	2.9673	down
hsa-miR-217	2.3688	up	1.2894	up	1.4012	up	1.5096	up
hsa-miR-151	2.6681	down	3.4184	down	1.2842	down	1.6828	down
hsa-miR-155	4.5868	down	4.2649	down	5.0020	down	2.3014	down
hsa-miR-193a	1.7291	down	5.2780	down	5.2598	down	1.1368	down
hsa-miR-559	2.1055	down	1.2961	down	1.8521	down	1.6984	down
hsa-miR-622	1.5263	up	2.6666	up	2.5580	up	1.5719	up
hsa-miR-183	2.5817	up	2.2127	up	3.9541	up	2.6268	up
hsa-miR-10a	1.0956	down	2.8073	down	7.8308	down	1.3441	down
hsa-miR-594	2.2894	up	1.3172	down	1.0052	up	1.5801	up
hsa-miR-488	1.2135	down	1.0064	down	1.0329	down	2.5565	down

hsa-miR-587	1.8943	down	1.0749	down	2.0753	up	1.1355	up
hsa-miR-4093p	1.8965	down	2.1710	down	1.1434	down	2.4005	down
hsa-miR-378	1.4331	down	2.8121	down	2.3565	down	2.4780	down
hsa-miR-21	1.2548	down	2.7463	down	9.2535	down	1.6994	down
hsa_SNORD3	1.6377	down	2.2333	down	1.7161	down	1.8266	down
hsa_SNORD15A	5.9278	down	4.6913	down	1.6217	down	8.8458	down
hsa-miR-222	2.6314	down	8.5940	down	3.8929	down	1.2775	down
hsa-miR-370	1.1434	down	2.5862	down	2.0789	down	1.9196	down
hsa-miR-221	4.2821	down	22.6405	down	5.8092	down	2.7431	down
hsa-miR-146a	7.0739	down	40.7153	down	65.3448	down	27.4741	down

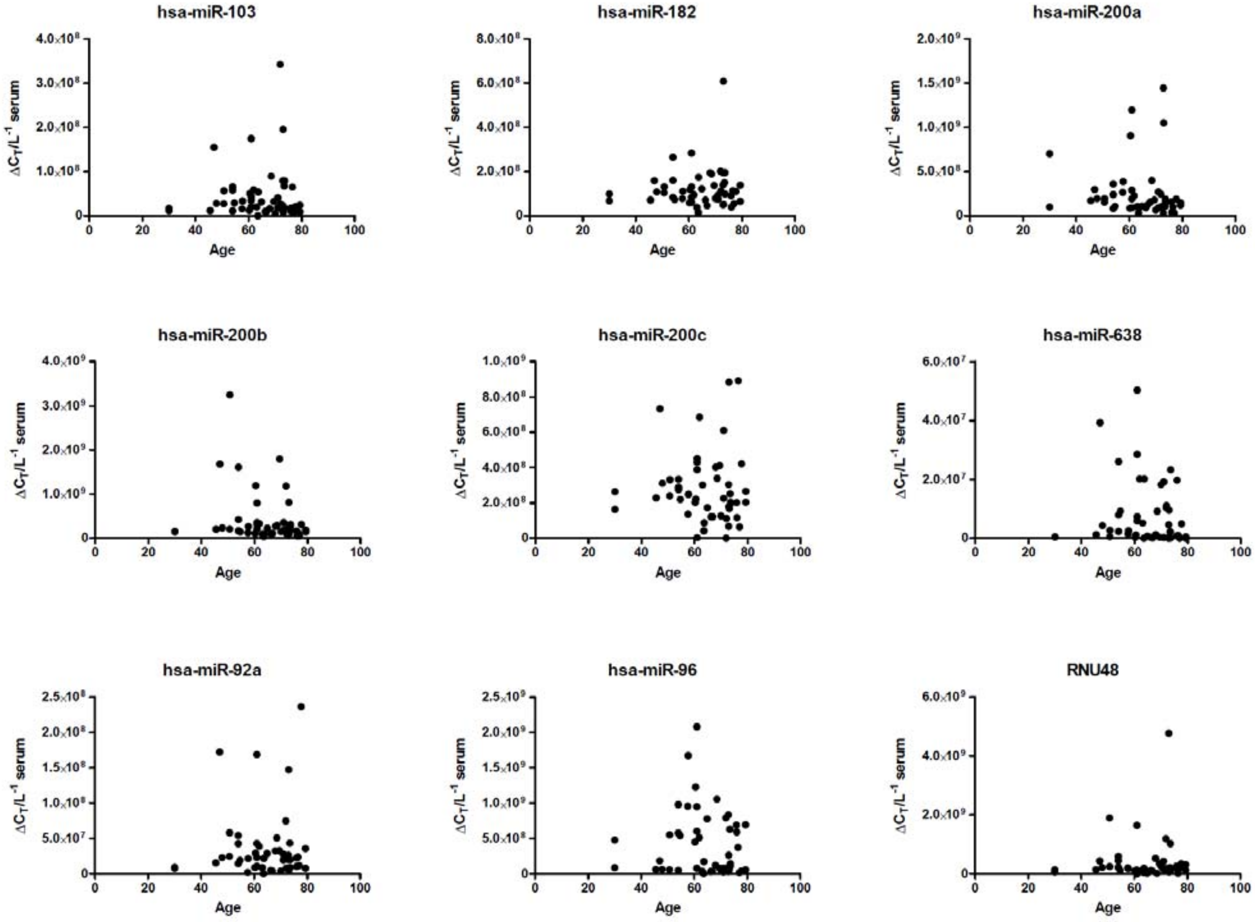

Figure S1. Serum miRNA levels relative to age

Volume adjusted values ($\Delta C_T/L^{-1}$ of serum assayed) are plotted relative to age (N=56).

Table S3. Pairwise correlation values for serum miRNA levels and CA-125, tumor size, progression free interval or overall survival. Volume adjusted miRNA values ($\Delta\Delta C_T/L^{-1}$ of serum assayed) normalized to miR-103 are correlated to pre-operative CA-125 measurements, tumor size (mm) and progression free interval and overall survival (months) (Pearson product-moment correlation coefficient (PCC); 2-tailed).

.

	CA-125 (N=14)		Tumor Size (N=20)		Progression Free Interval (N=14)		Overall Survival (N=20)	
miRNA	PCC	P	PCC	P	PCC	P	PCC	P
miR-92a	.116	.692	.051	.829	.061	.836	.087	.716
miR-638	.032	.913	.111	.643	-.037	.901	.082	.730
miR-200a	.247	.395	.302	.196	.415	.140	.160	.500
miR-200b	.120	.684	.205	.386	.332	.247	.121	.738
miR-200c	.215	.461	.335	.148	.192	.511	.073	.760
miR-182	.068	.818	.152	.523	.225	.439	-.017	.942
miR-96	.117	.690	-.165	.486	.001	.997	.115	.629
RNU48	-.042	.887	.136	.568	.372	.191	.092	.700
